# Favorable locoregional control in clinically node-negative hormone-receptor positive breast cancer with low 21-gene recurrence scores: a single-institution study with 10-year follow-up

**DOI:** 10.1186/s12885-022-10308-w

**Published:** 2022-11-25

**Authors:** Cihan Uras, Neslihan Cabioglu, Fatma Tokat, Ozlem Er, Halil Kara, Taner Korkmaz, Nuran Bese, Umit Ince

**Affiliations:** 1 Departments of Surgery, Acibadem Mehmet Ali Aydinlar University, School of Medicine, Istanbul, Turkey; 2 Department of Surgery, Istanbul University, Istanbul Faculty of Medicine, Istanbul, Turkey; 3 Department of Pathology, Acibadem Mehmet Ali Aydinlar University, School of Medicine, Istanbul, Turkey; 4 Department of Medical Oncology, Acibadem Mehmet Ali Aydinlar University, School of Medicine, Istanbul, Turkey; 5 Department of Radiation Oncology, Acibadem Mehmet Ali Aydinlar University, School of Medicine, Istanbul, Turkey

**Keywords:** Oncotype DX scores, Ki-67, Breast cancer, Histologic grade, Locoregional recurrence

## Abstract

**Background:**

Recent studies have shown a lower likelihood of locoregional recurrences in patients with a low 21-gene recurrence score (RS). In this single-institution study, we investigated whether there are any associations between different cutoff values of 21-gene RS, histopathological factors, and outcome in patients with long-term follow-up.

**Methods:**

The study included 61 patients who had early-stage (I-II) clinically node-negative hormone receptor-positive and HER2-negative breast cancer and were tested with the 21-gene RS assay between February 2010 and February 2013. Demographic, clinicopathological, treatment, and outcome characteristics were analyzed.

**Results:**

The median age was 48 years (range, 29–72 years). Patients with high histologic grade (HG), Ki-67 ≥ 25%, or Ki-67 ≥ 30% were more likely to have intermediate/high RS (≥ 18). Based on the 21-gene RS assay, only 19 patients (31%) received adjuvant chemotherapy. At a median follow-up of 112 months, 3 patients developed locoregional recurrences (4.9%), which were treated with endocrine therapy alone. Among patients treated with endocrine treatment alone (*n* = 42), the following clinicopathological characteristics were not found to be significantly associated with 10-year locoregional recurrence free survival (LRRFS): age < 40 years, age < 50 years, high histological or nuclear grade, high Ki-67-scores (≥ 15%, ≥ 20%, ≥ 25%, ≥ 30%), presence of lymphovascular invasion, luminal-A type, multifocality, lymph node positivity, tumor size more than 2 cm, RS ≥ 18, and RS > 11. However, patients with RS ≥ 16 had significantly poorer 10-year LRRFS compared to those with RS < 16 (75% vs. 100%, respectively; *p* = 0.039).

**Conclusions:**

The results suggest that patients with clinically node-negative disease and RS ≥ 16 are more likely to benefit from adjuvant chemotherapies. However, those with RS < 16 have an excellent outcome and local control in long-term follow-up with endocrine treatment alone.

## Background

Multigene prognostic gene assays are used to decide on the benefit of adjuvant chemotherapy in addition to endocrine treatment in early-stage, estrogen receptor (ER)-positive, HER2-negative breast cancer. The goal is to guide the personalized management in the contemporary era of breast-cancer treatment [1–6]. Particularly, the 21-gene recurrence score (RS) assay (Oncoype DX™, Genomic Health, Redwood City, CA) has been recommended by the 2022 guidelines of the National Comprehensive Cancer Network (NCCN) and is widely used in the USA [7].

The utility of this genetic assay has been validated in numerous clinical studies, including the Trial Assigning Individualized Options for Treatment (TAILORx) and RxPONDER trials on clinically lymph-node-negative and positive patients [8, 9]. The TAILORx trial reported no chemotherapy benefit in women who were older than 50 years of age, but in women who were 50 years of age or younger, adjuvant chemotherapy improved outcomes if the recurrence score was 16 to 25 [8]. Similarly, recent findings from the RxPONDER trial indicated that premenopausal women with node-positive disease and RS of 25 or lower have a better outcome with endocrine treatment in addition to chemotherapy compared to those with endocrine-treatment alone. Postmenopausal women with similar clinicopathological features did not show any benefit from adjuvant chemotherapy [9].

Previous studies demonstrated no benefit of adjuvant chemotherapy in patients with ER-positive/HER2-negative breast cancer classified as molecularly defined luminal A-type or immunohistochemically classified luminal A-type. These tumors have an excellent prognosis with a low Ki-67 index and progesterone receptor positivity [10–12]. However, due to the interobserver variability in assessment of the Ki-67 score [13, 14], its utility has been limited and is not accepted worldwide in tailoring the adjuvant treatment of early-stage hormone-receptor positive breast cancer.

A few recent reports have also shown a lower likelihood of locoregional recurrences in patients with a low 21-gene RS [15–18]. In this single-institution study, we investigated whether there are any associations of locoregional recurrence (LRR) with higher 21-gene RS in patients with early-stage ER-positive disease in long-term follow-up. We also investigated the associations of traditional biomarkers and histopathological factors with the Oncotype-DX recurrence RS.

## Methods

The study included consecutive female patients with early-stage clinically node-negative ER- positive breast cancer who were tested with the 21-gene RS assay and underwent surgery for breast cancer at the Acibadem Maslak Hospital with a follow-up of more than 5 years after surgery. Prospectively maintained data were retrospectively analyzed. Demographic, clinicopathological, treatment, and outcome characteristics were analyzed. The study was approved by the local institutional ethics committee at the Acibadem University, Faculty of Medicine.

Selected representative paraffine sections of the index tumor of the patients were studied by Genomic Health (Redwood City, CA, USA) to assess the Oncotype DX RS. The score was determined by 21-gene reverse transcriptase polymerase chain reaction (RT-PCR) using the RNA isolated from formalin-fixed paraffin-embedded tissue as described previously [19]. RT-PCR expression analysis (16 cancer-related genes and 5 reference genes) evaluates the expression of 16 cancer-related genes normalized by the expression of five reference genes. The result is a numeric value on a scale of 0 to 100.

Risk categories were determined according to RS as follows: a) low risk for scores < 18, b) intermediate risk for scores of 18–30, and c) high risk for scores ≥ 31, as previously reported [2]. Patients were also stratified into three risk categories based on different RS cutoff scores: low risk (RS < 11), intermediate risk (RS 11–25), and high risk (RS ≥ 26), as suggested previously [8].

The results of the 21-gene RS assay were prospectively incorporated in the treatment plan as recommended [2]. Most low-risk patients were treated with adjuvant endocrine therapy, whereas most high-risk patients received a combination of endocrine therapy and chemotherapy. The treatment of patients with intermediate RS was variable and depended on various clinicopathological features and individual choices. RS was not routinely considered in the selection of locoregional therapy. The results were correlated with histopathological factors including tumor size (≤ 2 cm vs > 2 cm), nuclear grade (NG), histological grade (HG), lymphovascular invasion (LVI), and different cutoff values of Ki-67 (≤ 10%, ≤ 15%, ≤ 20%, or ≤ 25%).

ER or PR positivity was considered for any nuclear immunohistochemistry (IHC) staining > 1%. HER2 positivity was determined by IHC and FISH findings, whereas Ki-67 scores were determined as suggested previously [13]. The tumor subtypes were defined as follows according to the IHC staining [20]: luminal A: ER( +) or PR( +), HER2-neu (-), Ki-67 < 20%; luminal B: ER( +) or PR( +), HER2-neu ( +) and/or Ki-67 ≥ 20%; non-luminal: HER2-neu( +), ER (-) PR(-) HER2-neu ( +); and triple-negative: ER(-) PR (-) HER2-neu (-).

Correlations were assessed between Oncotype DX expressions of ER, PR, or HER2, and IHC expressions of ER, PR, or HER2. Clinicopathological variables included patient age at breast-cancer diagnosis, tumor size, histological type of tumor, LVI, 21-gene RS result, local and systemic treatment, and clinical outcome. For multifocal/multicentric carcinomas, the size of the largest tumor and the highest RS result were recorded. For one patient with metachronous bilateral ER + /HER2 − breast carcinomas with low RS, only the data pertaining to the first tumor were included. The institutional database and electronic medical records were reviewed to record the date of last follow-up, date of death, and the date and type of LRR and distant recurrence.

### Statistical analysis

The statistical software program SPSS 25 (Statistical Package for Social Sciences; SPSS, IBM Corp., Armonk, NY, USA) was used for the statistical analyses. To assess the differences between the groups, categorical variables were evaluated by Pearson’s chi-squared and Fisher's exact tests in two-tailed univariate analyses. Overall survival was calculated from the date of pathological diagnosis of breast cancer to the date of last follow-up. Kaplan–Meier analyses were used for the survival curves test, and the log-rank test was used to compare different prognostics affecting the outcome, including different RS values.

Disease-free survival (DFS) was analysed based on local and systemic metastases, and disease-specific survival (DSS) rates were analysed based on breast-cancer-related mortality. LRR was defined as invasive breast cancer involving the ipsilateral breast parenchyma, axilla, regional lymph nodes, and chest wall and identified more than 6 months from the initial diagnosis of breast cancer. LRR-free survival was analysed by considering the locoregional metastases. Univariate association of the RS score with LRR-free survival was also examined among a subset of women treated with endocrine therapy and chemotherapy using as described above. *P*-values were two-sided, and a *p*-value equal to or less than 0.05 was considered as statistically significant.

## Results

The study included 61 consecutive female patients with early-stage breast cancer and clinically node negative disease between February 2010 and February 2013. The median age was 48 years (range, 29–72 years). Fifty-three patients were diagnosed with Stage I (86.9%), and 8 patients had Stage II (13.1%) following surgery. The majority of patients underwent breast-conserving surgery (BCS; *n* = 40; 65.6%) and sentinel lymph node biopsy (SLNB, *n* = 21, 34.4%). Of the 61 patients, 7 (11.5%) had axillary lymph-node positivity, and 6 patients underwent axillary dissection following SLNB. The demographic and clinicopathological characteristics of patients are shown in Tables 1 and 2.

**Table 1:** Clinicopathological characteristics of patients with clinically node-negative hormone receptor-positive HER2-negative breast cancer tested with 21-gene recurrence score assay

Clinicopathological Characteristics	N (%)
Median age (range, min–max)	48 (range, 29–72)
Premenopausal	35 (57.4%)
Postmenopausal	26 (42.6%)
Breast Surgery:	
Breast conservation	40 (65.6%)
Mastectomy	21 (34.4%)
Axillary surgery:	
SLNB	55 (90.2%)
SLNB&ALND	6 (9.8%)
Type of axillary metastasis:	
Isolated tumor cell (pN0_ITC_)	NA
Micrometastasis (pN_mic_)	3 (42.9%)
Macrometastasis (pN_1_)	4 (57.1%)
pT1	49 (80.3%)
pT2	12 (19.7%)
pN0	54 (88.5%)
pN1	7 (11.5%)
TNM Stage (7^th^ vs. 8^th^ version)	
Stage 1	44 (72.1%) vs. 53 (86.9%)
Stage 2	17 (27.9%) vs. 8 (13.1%)
Histopathology:	
Invasive ductal carcinoma	53 (86.9%)
Invasive lobular carcinoma	5 (8.2%)
Mucinous & tubular carcinoma	3 (4.9%)
ER positivity	61 (100%)
PR positivity	59 (96.7%)
Histologic Grade:	
Low	12 (19.7%)
Intermediate	39 (63.9)
High	10 (16.4%)
Nuclear Grade:	
Low	5 (8.2%)
Intermediate	40 (65.6%)
High	16 (26.2%)
LVI( +)	10 (17%)
Median Ki-67 index (%, range)	15 (range, 1–57)
Luminal-A (by IHC)	42 (68.9%)
Recurrence score:	
^a^ Low (< 18) / ^b^Low (< 11)	10 (16.4%) / 11 (18%)
^a^Intermediate (18–30) / ^b^Intermediate (11–25)	47 (77%) / 43 (70.5%)
^a^ High (≥ 31)/ ^b^High (> 26)	4 (6.6%) / 7 (11.5%)
Median follow-up	112 (range, 54–154)
Adjuvant chemotherapy	19 (31.1%)
Postmastectomy radiation	2 (9.5%)

*SLNB* Sentinel lymph node biopsy, *ALND* Axillary lymph node dissection, *IHC* Immunohistochemistry, *NA *Not available

^a ^[2]

^b ^[8]

**Table 2:** Correlation of 21-gene Recurrence score (RS) including different cut-off values with Ki 67 scores and histopathological factors

Characteristics	RS ≥ 11 (*n* = 50)	*p-value*	RS ≥ 18 (*n* = 25)	*p-value*	RS ≥ 26 (*n* = 7)	*p-value*	RS ≥ 31 (*n* = 4)	*p-value*
pT1(*n* = 49) vs. pT2 (*n* = 12)	41/49 (83.7%) vs. 9/12 (75%)	0.676	20/49 (40.8%) vs. 5/12 (41.7%)	0.999	5/49 (10.2%) vs 2/12 (16.7%)	0.615	4/49 (8.2%) vs. 0/12 (0%)	0.576
HG low & intermediate (*n* = 51) vs. high HG (*n* = 10)	40/51(78.4%) vs. 10/10 (100%)	**0.184**	16/51(31%) vs. 9/10 (90%)	**0.001**	3/53 (5.7%) vs. 2/8 (25%)	**0.080**	2/51 (3.9%) vs. 2/10 (20%)	0.122
NG low & intermediate (*n* = 45) vs high NG (*n* = 16)	35/45 (77.8%) vs. 15/16 (93.75%)	0.259	15/45 (33.3%) vs. 10/16 (62.5%)	0.074	1/45 (2.2%) vs. 6/16 (37.5%)	**0.001**	1/45 (2.2%)vs 3/16 (18.75%)	**0.05**
LVI (-) (*n* = 49) vs. LVI( +) (*n* = 10)	39/49 (79.6%) vs. 9/10 (90%)	0.670	17/49 (34.7%) vs. 6/10 (60%)	0.166	5/49 (10.2%) vs. 1/10 (10%)	0.999	3/49 (6.1%)vs 1/10 (10%)	0.534
Ki-67 < 15% (*n* = 29) vs. ≥ 15% (*n* = 32)	22/29 (75.9%) vs. 28/32 (87.5%)	0.323	10/29 (34.5%) vs. 15/32 (46.9%)	0.435	1/29 (3.4%) vs. 6/32 (18.75%)	0.106	1/29 (3.4%) vs. 3/32 (9.4%)	0.615
Ki-67 < 20% (*n* = 42) vs. ≥ 20% (*n* = 19)	33/42 (78.6%) vs. 17/19 (89.5%)	0.476	14/42 (33.3%) vs. 11/19 (57.9%)	0.094	3/42 (7.1%) vs. 4/19 (21%)	0.190	2/42 (4.8%) vs. 2/19 (10.5%)	0.582
**Ki-67 < 25% (*n* = 50) vs. ≥ 25% (*n* = 11)**	40/50 (80%) vs. 10/11 (90.9%)	0.670	17/50 (34%) vs. 8/11 (72.7%)	**0.038**	3/50 (6%) vs. 4/11 (36.4%)	**0.016**	2/50 (4%) vs. 2/11 (18.2%)	0.146
**Ki-67 < 30% (*n* = 53) vs. ≥ 30% (*n* = 8)**	42/53 (79.2%) vs. 8/8 (100)	0.330	18/53 (34%) vs. 7/8 (87.5%)	**0.006**	3/53 (5.7%) vs. 4/8 (50%)	**0.004**	2/53 (3.8%) vs. 2/8 (25%)	0.08

*RS* Recurrence score

The majority of the patients had invasive ductal carcinoma (81.6%) or tumors with low or intermediate nuclear (NG, *n* = 45, 73.8%) or histological grade (HG, *n* = 51, 83.6%). All tumors were hormone-receptor positive and HER2-negative, and the majority had no lymphovascular invasion (LVI, *n* = 49, 80.3%). The median Ki-67 score was 15% (range, 1%-57%). The associations between histopathological variables and different RS cutoff scores are shown in Table 2. Patients with high HG were more likely to have RS ≥ 18, whereas patients with high NG were more likely to have RS ≥ 26 or > 31. Similarly, patients with Ki-67 ≥ 25% or ≥ 30% were more likely to have RS ≥ 18 or ≥ 26. Briefly, patients with high HG, Ki-67 ≥ 25%, or Ki-67 ≥ 30% were more likely to have intermediate/high RS based on RS ≥ 18 (Table 2). The other associations did not reach the statistical significance.

### Outcome

Based on the 21-gene RS assay, only 19 patients (31%) received adjuvant chemotherapy, and 2 patients had postmastectomy radiation (9.5%). At a median follow-up of 112 months (range, 60–160 months), 3 patients developed locoregional recurrences (4.9%), which were treated with endocrine therapy alone. The clinicopathological characteristics of patients are shown in Table 3. Of these, two patients had locoregional recurrences including axillary and supraclavicular region with adjuvant radiation to the chest wall. The other patient had a local recurrence in the breast in a previously operated region of the primary tumor despite applying adjuvant radiation to the breast following surgery. All three patients had unifocal tumors with IHC classification as luminal A, ER and PR positivity > 50%, Ki-67 < 20%, and had locoregional recurrences at a median time of 105 months (range, 96–107 months) on long-term follow-up. Interestingly, all patients had RS ≥ 16, which is an intermediate score (11–25) according to the TAILOR-X and RxPONDER trials.

**Table 3:** Clinicopathological characteristics of patients with locoregional recurrence

Characteristics	Patient 1	Patient 2	Patient 3
Locoregional recurrence type	Breast recurrence	Supraclavicular LN metastasis	Axillary LN metastasis
Family history	No	No	No
Age	50	52	29
Surgery type	BCS&SLNB	BCS&SLNB	Mastectomy&SLNB
Surgical margin distance	10 mm	10 mm	4 mm
Tumor size/ pTNM	1.1 cm/ pT1N0	3 cm/ pT2N0	2 cm/ pT1N0
Histopathology	IDC	IDC	IDC
Histological/ Nuclear Grade	intermediate/intermediate	intermediate/intermediate	intermediate/intermediate
LVI	No	No	No
Multifocality/ EIC	No/No	No/No	No/Yes
ER rate /PR rate	100%/100%	95%/30%	95%/70%
Ki-67 score	10%	11%	18%
21-gene RS	21	17	17
Adjuvant Radiotherapy	Yes	Yes	Yes
Adjuvant Chemotherapy	No	No	No
Type of endocrine therapy	Tamoxifen/leuprolide acetate	letrozole	Tamoxifen/leuprolide acetate
LR time after surgery	98 months	107 months	105 months

*RS* Recurrence score, *LR* Locoregional recurrence, *LN* Lymph node, *BCS* Breast conserving surgery, *SLNB* Sentinel lymph node biopsy, *IDC* Invasive ductal cancer, *EIC* Extensive intraductal component

**Fig. 1:**
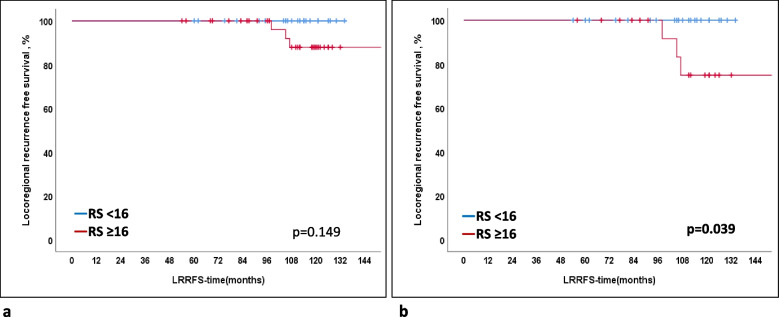
**a** Locoregional recurrence-free survival in patients with recurrence score (RS) < 16 (*n* = 25) vs. RS ≥ 16 (*n* = 36) in the whole cohort (*n* = 61, *p* = 0.149). **b** Locoregional recurrence-free survival in patients with RS < 16 (*n* = 25) vs. RS ≥ 16 (*n* = 17) without adjuvant chemotherapy (*n* = 42) (*p* = 0.039)

The 10-year DFS and LRRFS rates were 92.8%, whereas the 10-year DSS rate was 100% in the present cohort. Patients with RS ≥ 16 were found to have a poorer 10-year LRRFS at 88% compared to those with RS < 16 at 100% (*p* = 0.149, Fig. 1a). Among patients treated with endocrine treatment alone (*n* = 42), the clinicopathological characteristics not significantly found to be associated with 10-year LRRFS included age < 40 years, age < 50 years, high histological or nuclear grade, high Ki-67-scores (≥ 15%,, ≥ 20%, > 25%, > 30%), presence of lymphovascular invasion, luminal A type, multifocality, lymph node positivity, tumor size more than 2 cm, RS ≥ 18, and RS ≥ 11. However, patients with RS ≥ 16 had significantly poorer 10-year LRRFS and DFS (100% for RS < 16 vs. 75% for RS ≥ 16, *p* = 0.039), as demonstrated in Table 4 and Fig. 1b.

**Table 4:** Clinicopathological factors and 21-gene recurrence score (RS) associated with disease-free survival (DFS) and locoregional recurrence free survival free survival (LRRFS) (DFS is equal to LRRFS since none of the patients developed distant metastases)

Factors	10- year LRRFS = 10-year DFS	*p*-value
**Total (*n* = 61)**	92.8%	
**Recurrence Score:**		
RS ≤ 11 vs. RS ≥ 11	100% vs. 90.2%	0.292
RS < 16 vs RS ≥ 16	100% vs. 88%	0.149
RS < 18 vs. RS ≥ 18	91.6% vs. 94.1%	0.808
**Without Adjuvant Chemotherapy (*n* = 42)**	89.1%	
**Recurrence Score:**		
RS < 11 vs. RS ≥ 11	100% vs. 83%	0.180
RS < 16 vs. RS ≥ 16	100% vs. 75%	**0.039**
RS < 18 vs. RS ≥ 18	91.3% vs. 75%	0.274

## Discussion

Among various multigene prognostic gene assays, the 21-gene RS assay has been more widely used in patients who are classified as early stage according to AJCC TNM 8th edition, have ER + /HER2- breast cancer, or are to be spared from unnecessary adjuvant chemotherapies as recommended by the NCCN guidelines when detected with low RS [7]. The 21-gen RS assay was initially developed to assess the distant recurrence risk and was validated in patient samples from the from National Surgical Adjuvant Breast and Bowel Project (NSABP) trials B-14 and B-20 [2]. However, its clinical utility to estimate the locoregional recurrence risk is less studied so far and not well established in patients with long-term follow-up.

In this single-institution study with 10-year follow-up, we demonstrated that patients with early-stage ER-positive HER2(-) breast cancer, RS < 16, and mostly presenting with lymph node-negative breast cancer have excellent prognosis with endocrine treatment alone without any locoregional recurrence. However, patients with RS ≥ 16 were found to have a poorer 10-year DFS and LRRFS when treated with endocrine treatment alone, similar to other recent findings [8]. The TAILORx-study included only the clinically node-negative patients. Associations between various RS cutoff values and chemotherapy to improve the DFS have demonstrated some benefit in patients ≤ 50 years old with RS in the range of 16 to 25. Their 9-year DFS rate was 83.3% in a patient subgroup treated with endocrine treatment alone [8]. Notably, our study cohort included mostly premenopausal patients with a median age of 48 years and patients with RS ≥ 16. Thus, the population has relatively young age, is expected to have long-term survival, and are more likely to benefit from adjuvant chemotherapies.

Mamounas et al. first studied the association of locoregional recurrence with 21-gene RS assay scores in tamoxifen-treated patients with node-negative and ER-positive breast cancer in the NSABP B-14 and B-20 studies [15]. Of 895 tamoxifen-treated patients, significant associations were found between RS and LRR in patient cohorts of both trials, and RS was also demonstrated as an independent significant predictor of LRR in multivariate analysis. Similarly, Turashvili et al. further investigated the relationship between 21-gene RS and LRR risk among 2326 patients with node-negative ER + /HER2- breast cancer who were treated at the Memorial Sloan Kettering Cancer Center from 2008 to 2013 [16]. At a median follow-up of 53 months, patients with intermediate and high RS were more likely to have LRR compared to the patients with low RS. The hazard ratios were 2.81 (95% CI 1.41–5.56, *p* < 0.01) and 4.61 (95% CI 1.90–11.19, *p* < 0.01), respectively. A recent report from the same institution also indicated that extremely low LRR rates of 0.7% (8/1184) were observed among patients with a low RS < 18 treated with endocrine therapy alone [17]. However, Thaker et al. [21] could not find a correlation between ipsilateral breast tumor recurrence (IBTR) and RS, even though Ki-67 expression was significantly associated with both IBTR (*p* = 0.019) and RS (*p* = 0.002). Furthermore, an increased risk of locoregional recurrences was also demonstrated in node-positive ER-positive patients with high RS, which may justify postmastectomy radiation or more extensive radiation as an independent factor [22–25].

Finally, a recent meta-analysis including 16 studies with 21,037 patients investigated the association of RS with LRR in ER + /HER2- breast cancer with a mean follow-up of 66.4 months [18]. Patients with RS of 18–30 and RS > 30 were significantly more likely to develop LRR than those with RS < 18. The increased relative risks (RRs) were 1.76 (95% confidence interval (CI): 1.32–2.37) and 3.45, (95% CI: 2.63–4.53), respectively. Similarly, by using TAILORx cutoffs, those with RS ≥ 26 had an increased risk of LRR with an RR of 2.49 [95% CI: 0.68–9.39] compared to those with RS < 11.

In the present report, our study cohort included mostly patients with luminal A-type node-negative disease and a low Ki-67 index. In concordance with previous reports, our findings also have demonstrated that the 21-gene RS < 16 was the sole significant factor associated with the 10-year LRRFS among patients with good clinicopathological characteristics on long-term follow-up. The findings in the present study also indicated that patients with high HG or Ki-67 ≥ 25% were more likely to have intermediate/high RS based on RS ≥ 18, which is in concordance with some previous studies that similarly demonstrated a significant association between increasing RS and higher Ki-67 values [26–32]. However, although the Ki-67 proliferation value is important, it is not the sole determinant of the Oncotype DX score [26]. Furthermore, some tumors with a low RS may reveal surprisingly high Ki-67, whereas a small percentage of luminal-A-like tumors with a low Ki-67 index have RS > 25, and chemotherapy is recommended in such cases [27, 32]. Moreover, its clinical utility has been questioned due to the lack of consensus on scoring due to interobserver variability and cutoff value [20], and it has only “AJCC Level III” in the new AJCC edition. However, a major limitation of the present study is the relatively small number of patients in the cohort, despite the long-term follow-up. Therefore, we cannot conclude that tumor grade and Ki-67 may replace the 21-gene assay to help physicians to make an accurate decision chemotherapy use based on the findings of this study.

Due to the limited use of Ki-67 worldwide, alternative nomograms or alternative multivariable models called Magee Equations™ (ME) were developed to estimate the oncotype score at lower cost [31, 33, 34]. These models use routinely reported histopathology and breast-cancer biomarker data to provide a score similar to the oncotype score to be used in the “Magee Decision Algorithm™” along with the mitosis score [34]. Other models being developed to predict the 21-gene RS have alternatively included nomograms based on routine clinicopathological variables, including age, tumor size, tumor grade, PR status, LVI, and histological type of breast cancer [33]. However, further prospective studies are needed to validate whether these models could accurately replace the 21-gene RS assay.

## Conclusions

Our results suggest that patients with RS ≥ 16 are more likely to benefit from adjuvant chemotherapies in long-term follow-up to obtain excellent outcomes and local control. Larger prospective randomized studies are required to further investigate the clinical utility of RS testing to estimate the LRR risk and to establish better locoregional control in high-risk cases. Large prospective trials should study whether alternative models, including clinicopathological variables with lower cost, could replace the 21-gene RS for selected patients to make a decision about the adjuvant systemic treatment. Until then, genomic tests including the 21-gene RS remain as the gold-standard tools for determining the need for adjuvant chemotherapy in clinically node-negative HR( +) patients.

## Data Availability

The datasets used and/or analysed during the current study available from the corresponding author on reasonable request.
